# Epidemiology of extended-spectrum beta-lactamase-producing *Enterobacteriaceae* in an intensive care unit with no single rooms

**DOI:** 10.1186/s13613-017-0295-0

**Published:** 2017-07-03

**Authors:** Xavier Repessé, Margaux Artiguenave, Sophie Paktoris-Papine, Florence Espinasse, Aurélien Dinh, Cyril Charron, Faten El Sayed, Guillaume Geri, Antoine Vieillard-Baron

**Affiliations:** 1 Intensive Care Unit, Section Thorax-Vascular Disease-Abdomen-Metabolism, Assistance Publique-Hôpitaux de Paris, University Hospital Ambroise Paré, 9, Avenue Charles-de-Gaulle, 92100 Boulogne-Billancourt, France; 2 Infection Control Unit, Section Biology Pathology and Health Products, Assistance Publique-Hôpitaux de Paris, University Hospital Ambroise Paré, 92100 Boulogne-Billancourt, France; 3 Infectious Diseases Department, Assistance Publique-Hôpitaux de Paris, University Hospital Ambroise Paré, 92100 Boulogne-Billancourt, France; 4 Microbiology Unit, Section Biology Pathology and Health Products, Assistance Publique-Hôpitaux de Paris, University Hospital Ambroise Paré, 92100 Boulogne-Billancourt, France; 5 Faculty of Medicine Paris Ile-de-France Ouest, University of Versailles Saint-Quentin en Yvelines, 78280 Saint-Quentin-en-Yvelines, France; 6 INSERM U-1018, CESP, Team 5 (EpReC, Renal and Cardiovascular Epidemiology), UVSQ, 94807 Villejuif, France

## Abstract

**Background:**

The transmission of extended-spectrum beta-lactamase-producing *enterobacteriaceae* (ESBL) is prevented by additional contact precautions, mainly relying on isolation in a single room and hand hygiene. Contact isolation cannot be achieved in our 12-bed ICU, which has only double rooms. We report the epidemiology of ESBL imported, acquired and transmitted in an ICU with no single rooms.

**Methods:**

We prospectively conducted an observational and non-interventional study in a French 12-bed ICU. Inclusion criteria were patients >18 years of age treated by at least two successive nursing teams. Patient characteristics at admission and clinical data during hospital stay were collected prospectively. ESBL carriage was monitored using rectal swabs collected at admission and once weekly during the ICU stay. Potential cross-transmission was studied (1) by identifying index patients defined as possible ESBL sources for transmission, (2) by classifying each ESBL strain according to the cefotaximase *München* (CTX_M_) 1 and 9 groups and (3) by gene sequencing for remaining cases of possible transmission.

**Results:**

From June 2014 to April 2015, of 550 patients admitted to the ICU, 470 met the inclusion criteria and 221 had at least two rectal swabs. The rate of ESBL colonization, mainly by *Escherichia coli*, at admission was 13.2%. The incidence of ESBL acquisition, mainly with *E. coli* too, was 4.1%. Mortality did not differ between ESBL carriers and non-carriers. In univariate analysis, ESBL acquisition was associated with male gender, SAPS II, SOFA, chronic kidney disease at admission, duration of mechanical ventilation, need for catecholamine and the ICU LOS. In multivariate analysis, SAPS II at admission was the only risk factor for ESBL acquisition. We confirmed cross-transmission, emanating from the same index patient, in two of the nine patients with ESBL acquisition (0.8%, 2/221). No case of cross-transmission in the same double room was observed.

**Discussion and conclusion:**

Prevalence of ESBL colonization in our ICU was 13.2%. Despite the absence single rooms, the incidence of ESBL acquisition was 4.1% and cross-transmission was proven in only two cases, resulting from the same index patient who was not hospitalized in the same double room.

**Electronic supplementary material:**

The online version of this article (doi:10.1186/s13613-017-0295-0) contains supplementary material, which is available to authorized users.

## Background

Multidrug-resistant organisms (MDROs), and specifically third-generation cephalosporin-resistant *Enterobacteriaceae*, constitute a major problem in hospitals [1]. In the intensive care unit (ICU), Gram-negative resistant pathogens are responsible for longer hospitalizations and poorer outcomes [2, 3]. In the absence of new broad-spectrum antibiotics, there is a need to control antibiotic consumption and to prevent patient-to-patient cross-transmission. Recommended contact precautions, based on hand hygiene and the use of gowns, are considered as the cornerstone of preventive measures, along with isolation in a single room [4, 5], especially for known carriers of resistant bacteria, defined as methicillin-resistant *Staphylococcus aureus*, extended-spectrum beta-lactamase (ESBL) and non-fermenting Gram-negative bacilli resistant to many antibiotics [6]. Moreover, ESBL-producing *Enterobacteriaceae* have the particularity of being part of the digestive flora, which means that specific precautions are required for the disposal of stools [6]. However, all these preventive precautions have mainly been evaluated in the context of hospital outbreaks of ESBL-producing *K. pneumoniae* or *E. cloacae* [7]. They have become debatable for very uncommon outbreaks of ESBL-producing *E. coli*, and it has been suggested that routine contact isolation in a single room could be challenged in a non-epidemic setting [8, 9].

We studied the analytic epidemiology of ESBL acquisition and transmission in an ICU without single rooms and the capacity for strict isolation of patients. Our hypothesis was that an ICU with double rooms is not associated with unexpected high acquisition and cross-transmission of ESBL, providing that contact precautions are strictly applied. Our secondary objectives were to report the incidences of and factors associated with ESBL acquisition and colonization at admission.

## Methods

### Study population and data collection

This non-interventional observational and usual care study was prospectively conducted in the 12-bed ICU of the tertiary university hospital Ambroise Paré (Boulogne-Billancourt, France). As for all our non-interventional studies, patients or their relatives were routinely informed that data recorded during hospitalization in the ICU may be used for observational research and scientific publications and that they may refuse at any time.

Consecutive patients were included if they met the following inclusion criteria:

- Patients >18 years old admitted to ICU.
- Taken in charge by at least two shifts of nurses during their stay in our ICU.

Our nurses operate 12-h shifts and each cares for between two and three patients, as recommended by French law. Patients given nursing care for a single shift, i.e., who were discharged or who died in the first 12 h following admission, were not included.

We recorded the main characteristics at admission, as age, gender, Simplified Acute Physiology Score (SAPS II) [10], SOFA [11], hospitalization within the 3 months before ICU admission, antibiotic exposure and travel within the 3 months before admission, transfer from another service or institution, as well as the main information concerning the ICU stay (mechanical ventilation and duration, ICU length of stay [LOS], antibiotic prescription, duration of antibiotic exposure, central venous and arterial catheters, dialysis, in-ICU mortality).

### Hygiene and isolation protocol

Our 12-bed ICU has three units each of two double rooms, so that isolation of ESBL carriers in a single room cannot be achieved. Each double-room area is around 20 m^2^. A rigid removable curtain (H 1.5 m, L 1.75 m) separates the two beds in each room. A preventive isolation protocol with contact precautions is routinely implemented in our unit at admission for patients expected to have high-risk factors for antibiotic resistance (age > 65 years, transfer from another ward or institution or already known ESBL carriage). We routinely used waterless alcohol-based hand rub and wore gowns before entering the room for such patients. These preventive measures were stopped when the first sample showed no MDRO. Moreover, our institution implemented measures for the elimination of *excreta* of MDRO carriers, in particular the widespread use of bedpan liners (CareBag, Cleanis, Paris, France) and a specific washbasin for the scrubbing of the bedpans. Finally, the manager of the infection control unit of the hospital (FE) conducts weekly checks that contact precautions are applied, in collaboration with the nursing officer of the ICU.

Consumption of alcohol-based hand rub (expressed in L/1000 patient-days or mL/patient-day) and of antibiotics (expressed in defined daily doses/1000 patient-days) is evaluated each year, and compliance with the hand hygiene protocol is assessed using blinded audits. These audits check compliance with the protocol by means of two care bundles each relating to three aspects of hand hygiene, one before the nurse provides healthcare and one after.

### Microbiological analysis

Rectal swabs were sampled at admission and then weekly, every Monday, as recommended by our usual local protocol. They were sown on Drigalski medium (Biomérieux, Marcy l’Etoile, France) and then on ESBL selective medium, Chrom ID™ ESBL (Biomérieux, Marcy l’Etoile, France). After incubation at 35 °C, antimicrobial susceptibility was evaluated at 24 and 48 h for each colony that grew on the selective medium. The ESBL gene (*bla*) was amplified and analyzed by two polymerase chain reactions for a cefotaximase *München* (CTX_M_) 1 and 9 groups. The gene *bla* was sequenced according to the Sanger method on the capillary sequencer ABI Prism 3130 (Applied Biosystems, Villebon-sur-Yvette, France) with the kit BigDye Terminator Cycle Sequencing (Thermo Fisher Scientific, Villebon-sur-Yvette, France). Comparison with already known sequences was made with the BLAST program (Basic Local Alignment Search Tool, blast.ncbi.nlm.nih.gov) from the National Center for Biotechnology Information.

ESBL acquisition was defined as a negative first screening at admission with a positive one during the ICU stay, at least 48 h after admission. ESBL colonization was defined as a positive ESBL screening at admission. All acquired carriers were considered as possible cases of patient-to-patient transmission. Patient-to-patient transmission was analyzed in three steps. The first step was the identification of potential index patients defined as known ESBL carriers hospitalized at the same time as the newly acquired carrier. The second step was the description and identification of ESBL as well as their comparison according to their CTX_M_ 1 or 9 groups. Transmission was excluded if the index’s ESBL strains differed from the case’s. Finally, the third step was the sequencing of the genes (*bla*) corresponding to the matched identified group. Transmission was excluded if the gene sequencing differed. Transmission was retained in the case of a matched ESBL group in two patients who had been hospitalized in the same period for at least one day if the gene sequencing was similar.

### Statistical analysis

Categorical variables were described as *n* (%) and compared with Pearson’s Chi-squared test or Fisher’s exact test, as appropriate. Continuous variables were described as median [interquartile] and compared using a Mann–Whitney test.

Factors associated with ESBL acquisition were picked up using a univariate analysis. Then, clinically relevant factors significantly associated with ESBL acquisition in the univariate analysis were included in a multivariate logistic regression. ESBL acquisition was the dependent variable. Regarding the nonlinear relationship between ICU LOS, SAPS II and outcome, we used fractional polynomials (using the *mfp* Stata function) for a better fit between independent and dependent variables, as previously recommended [12]. We presented the results as the odds ratio of ESBL acquisition for the median of categories (named as reference), as previously reported [12]. The goodness of fit of the model was studied using the Hosmer–Lemeshow test. Statistical analysis was performed using MedCalc Software™, Ostend, Belgium. The multivariate logistic regression was performed using Stata 14.1 software (Stata Corp., College Station, TX, USA). A *p* value < 0.05 was considered as statistically significant.

## Results

### Settings and characteristics of the population

**Fig. 1:**
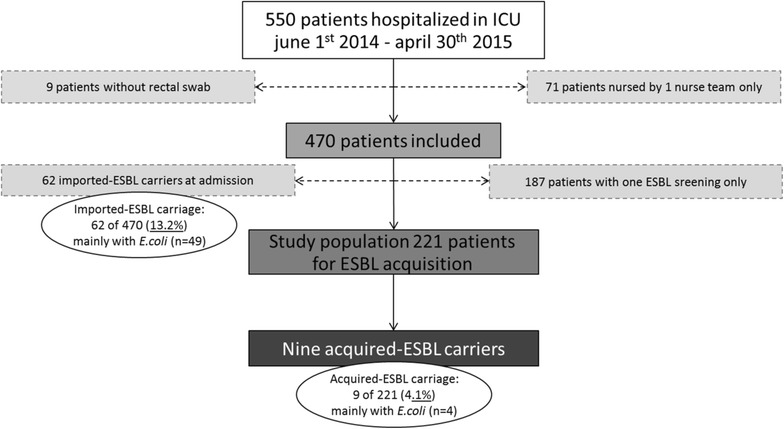
Flowchart of the patients included in the study. *ESBL* extended-spectrum beta-lactamase, *ICU* intensive care unit

Of 550 patients admitted to the ICU between June 1, 2014, and April 30, 2015, 71 were not included because they were present in the ICU for only one 12-h nursing shift. Nine patients were excluded because no rectal swab was sampled at admission. Finally, 470 patients met the inclusion criteria (Fig. 1). Their mean age was 66.0 [54.0, 77.0] years, 60.2% (*n* = 283) were males, and their mean SAPS II was 46.0 [32.0, 62.0] (Table 1). The hospitalization LOS was 4.0 [2.0, 8.0] days, and duration of mechanical ventilation was 2.0 [0.0, 5.0] days. Two hundred and twenty-four patients (47.8%) received mechanical ventilation. Half of the patients received catecholamine and had at least one central venous catheter and/or an arterial catheter. In-ICU mortality was 13.2%.

**Table 1:** Main characteristics of the population

Variable	All patients *n* = 470	No ESBL colonization *n* = 408	ESBL colonization *n* = 62	*p*
Age (years)	66.0 [54.0, 77.0]	66 [54, 77]	68 [57, 78]	0.5
Age > 65 years	256 (54.5)	218 (53.4)	38 (61.3)	0.3
Male gender	283 (60.2)	245 (60.0)	38 (61.3)	0.9
SAPS II	46.0 [32.0, 62.0]	45 [32, 62]	49 [37.3, 55.5]	0.5
SOFA score	7.0 [4.0, 9.0]	6 [4, 9]	7 [4.5, 8.5]	0.6
Preexisting conditions				
Heart failure	65 (13.9)	53 (13.0)	12 (19.3)	0.2
Peripheral arterial disease	60 (12.8)	45 (11.0)	15 (24.2)	0.007
Diabetes	110 (23.5)	91 (22.3)	19 (36.8)	0.2
Cirrhosis	21 (4.5)	18 (4.4)	3 (4.8)	0.8
Chronic obstructive pulmonary disease	69 (14.7)	59 (14.4)	10 (16.1)	0.9
Chronic kidney disease	61 (13.0)	52 (12.7)	9 (14.5)	0.8
Immunosuppression	75 (16.0)	62 (15.2)	12 (19.3)	0.5
Mechanical ventilation	283 (60.2)	248 (60.8)	35 (56.5)	0.6
Catecholamine use	224 (47.8)	190 (46.6)	34 (54.8)	0.3
Duration of mechanical ventilation (days)	2.0 [0.0, 5.0]	2.0 [0.0, 5.0]	1.0 [0.0, 4.0]	0.4
ICU length of stay (days)	4.0 [2.0, 8.0]	4 [2.0, 8.0]	4.0 [3.0, 8.0]	0.8
Transfer from another unit	239 (50.9)	206 (50.5)	33 (53.2)	0.8
Postoperative care	109 (23.2)	94 (23.0)	14 (22.6)	0.9
Hospitalization within the previous 3 months	243 (51.7)	204 (50)	39 (62.9)	0.08
Antibiotics within the previous 3 months	81 (17.2)	68 (16.7)	13 (20.1)	0.5
Antibiotics during ICU	346 (73.6)	303 (74.3)	43 (69.3)	0.5
Duration of antibiotic therapy	3.0 [0.0, 6.0]	3.0 [0.0, 6.0]	3.0 [0.0, 7.0]	0.8
Mortality	62 (13.2)	56 (13.7)	6 (9.7)	0.2

Categorical data are expressed as *n* (%)

Quantitative data are expressed as median [IQR]

The consumption of alcohol-based hand rub was 136.8 L/1000 patient-days in 2014 and 135.0 L/1000 patient-days in 2015. The consumption of antibiotics was 1453 DDD/1000 patient-days and 1331 DDD/1000 patient-days in 2014 and 2015, respectively. The blinded audits showed 63% compliance in 2014 and 77% in 2015.

### ESBL-imported carriage

Of the 470 patients included, 62 (13.2%) carried an ESBL at ICU admission, with *Escherichia coli* in 77.9% of the strains (Table 2). The main characteristics of these patients are presented in Table 1. Four of these 62 patients were colonized with two strains of ESBL and one with three strains. As given in Table 1, no factor associated with ESBL-imported carriage, except a history of peripheral arterial disease (*p* = 0.007), was observed in univariate analysis, in particular age (*p* = 0.8), SAPS II (*p* = 0.6), hospitalization during the past three months (*p* = 0.08), transfer from another unit (*p* = 0.8) and antibiotic exposure during the past three months (*p* = 0.5).

**Table 2:** Summary of the 68 strains responsible for the 62 ESBL-imported carriages and of the ten strains responsible for the nine ESBL-acquired carriages

Strain	Imported ESBL				Acquired ESBL			
	*n*	CTX_M_ 1 group	CTX_M_ 9 group	Other group	*n*	CTX_M_ 1 group	CTX_M_ 9 group	Other group
*E. coli*	53	30	15	8	5	3	2	0
*K. pneumoniae*	10	8	0	2	1	1	0	0
*E. cloacae*	1	1	0	0	2	2	0	0
*C. freundii*	1	1	0	0	1	0	0	1
*P. mirabilis*	0	0	0	0	1	1	0	0
*P. vulgaris*	1	0	0	1	0	0	0	0
*C. koseri*	2	2	0	0	0	0	0	0
Total	68	43	15	11	10	7	2	1

### ESBL acquisition

The incidence of ESBL acquisition was analyzed in 221 patients, whose characteristics are presented in Table 3. Nine patients acquired ESBL carriage, leading to an overall incidence of 4.1%. One of them acquired two different ESBL strains. The median time of acquisition was 8 [5–11] days. As reported in Table 3, in univariate analysis, those patients had higher SAPS II (*p* = 0.007) and SOFA (*p* = 0.01) scores, with a longer LOS (11 [9, 26] vs. 7 [5, 12] days, respectively, *p* = 0.007), than patients who did not acquire ESBL. All of them received mechanical ventilation (*p* = 0.1) and catecholamine infusion (*p* = 0.001). In multivariate analysis, only SAPS II at admission remained associated with ESBL acquisition (Table 4).

**Table 3:** Main characteristics of the study population for ESBL acquisition and factors associated with ESBL acquisition in univariate analysis

Variable	All patients *n* = 221	No ESBL acquisition *n* = 212	ESBL acquisition *n* = 9	*p*
Age (years)	67.0 [55.0, 77.0]	66.5 [55.0, 76.0]	82.0 [66.0, 82.0]	0.07
Age > 65 years	123 (55.6)	116 (54.7)	7 (78)	0.3
Male gender	144 (65.2)	137 (64.6)	7 (77.8)	0.5
SAPS II	49.0 [35.0, 66.0]	48.0 [35.0, 65.0]	72.0 [55.0, 77.0]	0.007
SOFA score	7.0 [4.0, 10.0]	7.0 [4.0, 10.0]	10.0 [9.0, 12.0]	0.01
Preexisting condition				
Heart failure	32 (14.5)	30 (14.1)	2 (28.6)	0.6
Peripheral arterial disease	25 (11.3)	22 (10.3)	3 (33.3)	0.07
Diabetes	54 (24.4)	51 (24)	3 (33.3)	0.5
Cirrhosis	10 (4.5)	9 (4.2)	1 (11.1)	0.4
Chronic obstructive pulmonary disease	39 (17.6)	37 (17.5)	2 (22.2)	0.7
Chronic kidney disease	36 (16.3)	32 (15.1)	4 (44.4)	0.04
Immunosuppression	38 (17.2)	36 (17.0)	2 (22.2)	0.7
Mechanical ventilation	166 (75.1)	157 (74.1)	9 (100)	0.1
Catecholamine use	132 (59.7)	123 (58.0)	9 (100)	0.01
Duration of mechanical ventilation (days)	4.0 [1.0, 10.0]	4.0 [0.0, 10.0]	9.0 [7.0, 11.0]	0.03
ICU length of stay (days)	8.0 [5.0, 12.0]	7.0 [5.0, 12.0]	11.0 [9.0, 26.0]	0.007
Transfer from another unit	124 (56.1)	117 (55.2)	7 (77.8)	0.3
Postoperative care	58 (26.2)	54 (25.5)	4 (44.4)	0.2
Hospitalization within the previous 3 months	123 (55.7)	117 (55.2)	6 (66.7)	0.7
Antibiotics within the previous 3 months	40 (18.1)	37 (17.5)	3 (33.3)	0.2
Antibiotics during ICU stay	193 (87.3)	184 (86.8)	9 (100)	0.6
Duration of antibiotic therapy	5.0 [3.0, 8.0]	5.0 [3.0, 8.0]	8.0 [5.0, 13.5]	0.1

Categorical data are expressed as *n* (%)

Quantitative data are expressed as median [IQR]

**Table 4:** Multivariate analysis of factors associated with ESBL acquisition

Variable	Reference	Odds ratio	95% Confidence interval
ICU length of stay (days)			
<4	3	0.92	0.82, 1.04
4–7	6	1.00	1.00, 1.00
7–10	9	1.08	0.96, 1.22
>10	15	1.28	0.89, 1.83
SAPS II			
<32	25	1.00	1.00, 1.00
33–45	40	1.89	1.11, 3.24
45–60	53	3.44	1.22, 9.68
>60	73	7.40	1.38, 39.59

Multivariate logistic regression including 218 complete observations

Hosmer–Lemeshow *p* value 0.6

SAPS II and ICU length of stay were included as continuous variables

SAPS II was not transformed

ICU length of stay was included using the following equation *x* ^2^ − 1.96 where *x* = (los)/10

Covariates were included as continuous variables in the multivariable model

Presented odds ratios were calculated for the reference indicated in the table

### ESBL cross-transmission

**Fig. 2:**
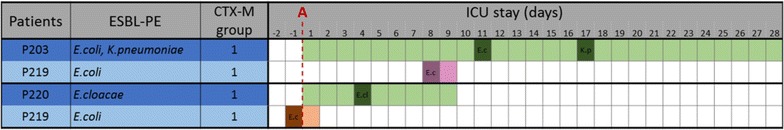
Chronological scale of the two cases of ESBL cross-transmission in the ICU. The figure represents the timescale of ESBL cross-transmission. Patients are identified with their inclusion number. The two cases of cross-transmission (P203 and P220) are colored in *dark blue*, whereas the corresponding index patient (P219) is materialized in *light blue*. Index patient is defined as already known carriers of ESBL who shared at least one day of hospitalization with the case of ESBL acquisition. *Each line* represents the stay of one patient. A day of hospitalization is represented by *a square*. *Each block* separated by *bold lines* represents a case of cross-transmission. The ICU stays of the cross-transmitted ESBL patients are *green*, whereas the stay of the index patient is colored in *pink* if the index patient is hospitalized in a different unit, in *orange* for hospitalization in the same unit but not in the same room. The *dotted red line* indicates the arrival in the ICU of patients acquiring ESBL. *A* arrival, *Ec Escherichia coli*, *Ecl Enterobacter cloacae*, *Cf Citrobacter freundii*, *CTXm* cefotaximase München, *ESBL-PE* extended-spectrum beta-lactamase-producing *Enterobacteriaceae*, *ICU* intensive care unit, *Kp Klebsiella pneumoniae*, *P* patient, *Pm Proteus mirabilis*

All acquired ESBL carriers were considered as possible cases of transmission. The nine patients with ESBL acquisition were associated with 16 potential index patients for whom the chronological scale analysis is reported in Additional file 1. Of five strains of *E. coli* isolated, three belonged to the group CTX_M_ 1 and 2 to the CTX_M_ 9 group (Table 2). The five other ESBL were mainly from the CTX_M_ 1 group (*n* = 4), and we were unable to identify the group in one case (*Citrobacter freundii*). The ESBL group excluded cross-transmission in four cases. For the five remaining acquired ESBL carriers, with the strain belonging to the same group as the potential index patient, gene sequencing identified the same gene for three patients (index P219 for two cases P203 and P220, Fig. 2). For these two cases of acquired ESBL (P203 and P220), patient-to-patient transmission was considered as proven. The acquired gene was observed in *E. coli* in one case (P203) and in *E. cloacae* in the other (P220). Both patients who acquired ESBL shared, respectively, one and two days with the index patient (Fig. 2). Neither was hospitalized in the same double room as the index patient: One patient was hospitalized in the same unit and the other in another one.

## Discussion

In this prospective observational study in 470 consecutive patients admitted to a 12-bed medical and surgical ICU with no single rooms, the prevalence of imported ESBL carriage was 13.2% and the incidence of acquired carriage was 4.1%, mostly with *E. coli*. No factor was identified as associated with imported ESBL carriage, while the severity at admission (SAPS II) was independently associated with ESBL acquisition. Only two cases of cross-transmission from the same index patient were reported.

### Imported carriage

The rate of imported ESBL carriage reported here is comparable to the 10–15% previously reported in three studies performed in French ICUs [13–15]. This prevalence is much higher than the 3.5% recently reported by Barbier et al. [16], but the latter study was performed in a historical cohort (1996–2016) of 16,734 patients admitted to 17 French ICUs and ESBL prevalence has changed dramatically in the last two decades. Although we noted no relation between severity at admission and imported ESBL, Alves et al. recently reported a lower rate of imported ESBL carriage of 8% in 308 patients with a much lower severity than in our population [17]. Finally, like Razazi et al. [13], we did not identify any impact of the ESBL-imported carriage on mortality, confirming the data of Barbier et al., who showed that ESBL infection was responsible for a 1.8-fold increase in mortality, while ESBL carriage had no impact on mortality [16].

### Acquired carriage

Despite the unfavorable double-room configuration of our ICU, the 4.1% rate of ESBL acquisition was much lower than the 13% reported by Razazi et al. in a 24-bed ICU with eight single rooms but without any protocol of contact precautions for ESBL carriers [13]. It is close to that reported by Alves et al. in an ICU with only single rooms, in which contact precautions were also applied [17]. Unlike Barbier et al., who reported that half of the ESBL carriers acquired their ESBL during their ICU stay [16], and Gardam et al., who reported that ESBL acquisition accounted for two-thirds of ESBL carriage in the ICU [18], ESBL acquisition accounted for only 12.7% of all ESBL carriage in our study, confirming that ESBL carriage is mostly imported, whereas high-level cephalosporinase (HL-Case) is mostly acquired, in the ICU [19]. In multivariate analysis, the severity (SAPS II) at admission was the only factor identified to be associated with the acquired carriage of ESBL, while some authors have reported that the duration of exposure to an ESBL carrier is independently associated with ESBL acquisition [13, 20]. Nevertheless, we found very short contact times for both cases of proven cross-transmission and ICU LOS was not independently associated with acquisition of ESBL in our study, which seems to temper the impact of the duration of contact in the mechanism of ESBL acquisition.

Our results confirm our hypothesis that a high level of infection control and a low rate of cross-transmission can be achieved despite the absence of isolation in single rooms, providing compliance with hand hygiene is high and antibiotic consumption is controlled. Consumption of alcohol-based hand rub for the study period was twice that usually reported in European ICUs [21], and the results of blinded audits of hand hygiene were considered very acceptable. Zahar et al. suggest that contact isolation is not needed to control the spread of ESBL-producing *E. coli* [9]. This was recently confirmed by Tschudin-Sutter et al. who showed no increase of transmission of ESBL-producing *E. coli* after contact precautions were discontinued, neither in an acute-care setting, nor in a geriatric hospital [20]. Moreover, Derde et al. demonstrated that 80% compliance with hand hygiene was associated with a decrease in *S. aureus* (MRSA) acquisition, even if it was not proven for vancomycin-resistant *Enterococci* (VRE) or highly resistant *Enterobacteriaceae* [22]. Guidelines concerning contact precautions and the recommended isolation rely on studies demonstrating their efficacy in controlling the spread of MRSA [23], VRE [24] and ESBL-producing *K. pneumoniae* [7], but such measures have not been fully validated for the now predominant CTX_M_ group of ESBL-producing *E. coli*. Isolation in single rooms could raise other issues since it has been shown to be associated with adverse outcomes, such as less patient–healthcare worker contact, delays in care provided, increased non-infectious adverse events and increased symptoms of depression and anxiety in patients [25].

### Cross-transmission

Only two cases of proven cross-transmission were identified among the nine acquired ESBL carriers. Both transmissions emanated from the same index patient and did not occur in the same double room. These results are in accordance with former studies that showed that cross-transmission is quite a rare mechanism of ESBL acquisition. In a Swiss population including ICU and non-ICU patients, Tschudin-Sutter et al. [8] confirmed transmission by pulse-field gel electrophoresis in two (1.5%) of 133 contacts of ESBL carriers hospitalized in the same room at least 24 h before identification of ESBL carriage and then isolation in a single room. Among 19 acquired ESBL carriers, Alves et al. recently identified only one case of likely patient-to-patient cross-transmission [17]. In a study at the beginning of the 2000s in a transplantation unit of four rooms with four beds, eight double rooms and four single rooms, Gardam et al. reported possible patient-to-patient transmission in only six of 69 cases of ESBL acquisition [18]. In fact, the mechanism of acquisition of ESBL is not so obvious and the antibiotic exposure or the persistence of pathogens on inanimate surfaces could play an important role [26], even if the role of persistent environmental contamination as a reservoir for ESBL cross-transmission remains debatable. While *A. baumannii* is well known to survive in surface dust for months, other Gram-negative bacteria are not usually resilient to desiccation, while *E. coli*, *Klebsiella* spp. and *P. aeruginosa* are reportedly also able to survive more than a year on inert surfaces [26].

### Limitations

We acknowledge that our study suffers from several limitations. First, the rate of acquisition could have been underestimated. Indeed, we did not perform a rectal swab at discharge and some patients may have acquired an ESBL between the last rectal swab and ICU discharge. Moreover, the number of patients could be considered as insufficient since 187/470 patients were not included in the analysis of the acquisition because they had only one rectal swab. Finally, it could be assumed that some cases of acquisition were not detected because of the lack of sensitivity of the rectal screening, in particular in patients with low levels of microorganisms, since we did not use enrichment before plating, which has been reported to improve sensitivity [27]. Nevertheless, several measures were applied to limit the number of false-negative screening results. First, nurses were trained to send for analysis only samples with a sufficient amount of stool on the swab. Second, the laboratory asked for a new sample if the standard cultures were sterile. Finally, swabs were seeded on a selective medium to improve the detection of ESBL. The second limitation is that our study does not allow understanding the mechanisms of acquisition when cross-transmission was excluded. We cannot rule out other hypotheses, such as transmission from unidentified carriers at admission, because of a lack of sensitivity of the rectal ESBL screening, and such as the impact of the selection pressure of antibiotics or the role of contamination by health care workers or inanimate surfaces.

## Conclusion

We reported an ESBL colonization prevalence of 13.2% at ICU admission, mainly with *E. coli*, and an incidence of ESBL acquisition of 4.1%, among which we noted only two cases of ESBL cross-transmission. Our study demonstrates that, despite the lack of single rooms, it is possible to reach a high level of infection control and a low rate of cross-transmission. The impact of isolation in the prevention of ESBL cross-transmission in the absence of an epidemic setting and the mechanisms underlying ESBL acquisition remain to be elucidated.

## Additional file

**Additional file 1.** The figure represents the timescale of ESBL acquisition. Patients are identified with their inclusion number. The nine patients who acquired ESBL during are colored in *dark blue*, whereas the 16 corresponding index patients are materialized in *light blue*. Index patients are defined as already known carriers of ESBL who shared at least one day of hospitalization with a case of acquisition. Among the 16 potential index patients, two were newly acquired ESBL carriers (P203 and P220). Eight index patients were hospitalized in the same unit and seven in a different one. Only one potential index patient was hospitalized in the same room. The median time of acquisition was 8 [5–11] days, while the median shared hospitalization with the index patients was 5 [3–9] days. *Each line* represents the stay of one patient. A day of hospitalization is represented by *a square*. *Each block* separated by *bold lines* represents a case of acquisition. The ICU stays of the patients who acquired ESBL are *green*, whereas the stay of the index patients is colored in *pink* if the index patient is hospitalized in a different unit, in *orange* for hospitalization in the same unit but not in the same room and in *yellow* for hospitalization in the same unit and the same room. The *dotted red line* indicates the arrival in the ICU of patients acquiring ESBL. The cases of transmission and their relatives’ index patients are separated by a *horizontal black bold line*. *A* arrival, *Ec Escherichia coli*, *Ecl Enterobacter cloacae*, *Cf Citrobacter freundii*, *CTXm* cefotaximase München, *ESBL-PE* extended-spectrum beta-lactamase-producing *Enterobacteriaceae*, *ICU* intensive care unit, *Kp Klebsiella pneumoniae*, *P* patient, *Pm Proteus mirabilis*.

## Abbreviations

ESBL: extended-spectrum beta-lactamase
CTX_M_: cefotaximase *München*
ICU: intensive care unit
LOS: length of stay
MDRO: multidrug-resistant organism
SAPS II: Simplified Acute Physiology Score
SOFA: Sequential Organ Failure Assessment
