# Supplementary figures and images for: Epidemiology of extended-spectrum beta-lactamase-producing Enterobacteriaceae in an intensive care unit with no single rooms
Source: Ann Intensive Care. 2017 Jul 3;7:73. doi: 10.1186/s13613-017-0295-0 (PMC5495817; doi:10.1186/s13613-017-0295-0)

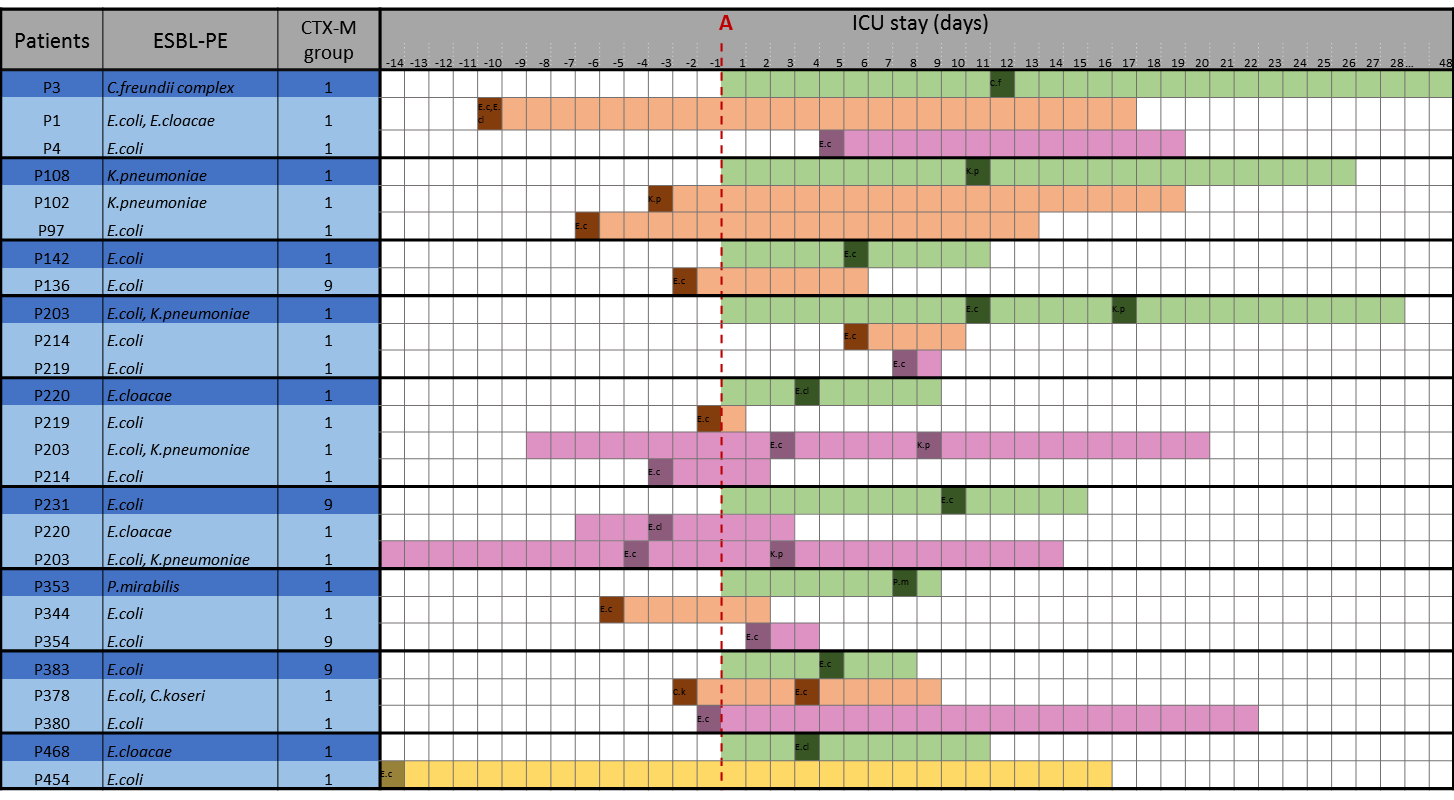

Supplement: Supplementary file 1 — Additional file 1. The figure represents the timescale of ESBL acquisition. Patients are identified with their inclusion number. The nine patients who acquired ESBL during are colored in dark blue, whereas the 16 corresponding index patients are materialized in light blue. Index patients are defined as already known carriers of ESBL who shared at least one day of hospitalization with a case of acquisition. Among the 16 potential index patients, two were newly acquired ESBL carriers (P203 and P220). Eight index patients were hospitalized in the same unit and seven in a different one. Only one potential index patient was hospitalized in the same room. The median time of acquisition was 8 [5–11] days, while the median shared hospitalization with the index patients was 5 [3–9] days. Each line represents the stay of one patient. A day of hospitalization is represented by a square. Each block separated by bold lines represents a case of acquisition. The ICU stays of the patients who acquired ESBL are green, whereas the stay of the index patients is colored in pink if the index patient is hospitalized in a different unit, in orange for hospitalization in the same unit but not in the same room and in yellow for hospitalization in the same unit and the same room. The dotted red line indicates the arrival in the ICU of patients acquiring ESBL. The cases of transmission and their relatives’ index patients are separated by a horizontal black bold line. A arrival, Ec Escherichia coli, Ecl Enterobacter cloacae, Cf Citrobacter freundii, CTXm cefotaximase München, ESBL-PE extended-spectrum beta-lactamase-producing Enterobacteriaceae, ICU intensive care unit, Kp Klebsiella pneumoniae, P patient, Pm Proteus mirabilis. [file 13613_2017_295_MOESM1_ESM.tif]
